# Primary Stroke Screening and Hydroxyurea Treatment for Sickle Cell Anemia in Pediatric Healthcare Settings in East and Central Africa: A Narrative Review of Capacity Gaps and Opportunities

**DOI:** 10.3389/phrs.2025.1608359

**Published:** 2025-05-15

**Authors:** Teresa Smith Latham, Katarzyna Czabanowska, Suzanne Babich, Faith Yego-Kosgei, Lisa M. Shook, Russell E. Ware

**Affiliations:** ^1^ Cincinnati Children’s Hospital Medical Center, Cincinnati, OH, United States; ^2^ College of Medicine, University of Cincinnati, Cincinnati, OH, United States; ^3^ Global Health Center, Cincinnati Children’s Hospital Medical Center, Cincinnati, OH, United States; ^4^ Care and Public Health Research Institute, Maastricht University, Maastricht, Netherlands; ^5^ Richard M. Fairbanks School of Public Health, Indiana University, Purdue University Indianapolis, Indianapolis, IN, United States; ^6^ Department of Health Policy Management and Human Nutrition, School of Public Health, Moi University, Eldoret, Kenya

**Keywords:** sickle cell anemia, stroke, hydroxyurea, resource-limited settings, capacity building

## Abstract

**Objectives:**

Sickle cell anemia (SCA) is associated with increased morbidity and mortality and impacts resource-limited settings with limited capacity for diagnosis and treatment. This review provides context for the magnitude of the problem, describes screening methods to prevent stroke, and factors that impact outcomes.

**Methods:**

A narrative review was conducted. Topics included background information on SCA, its clinical characteristics, complications including primary stroke, and available treatment options. Social, economic, and political factors in East and Central Africa were described.

**Results:**

A total of 37 publications were categorized into four themes: morbidity and mortality of SCA in sub-Saharan Africa; TCD screening for risk of primary stroke in children; treatment of children with SCA in resource-limited settings; and approaches to capacity gaps.

**Conclusion:**

SCA represents a public health problem in sub-Saharan Africa. TCD screening with hydroxyurea treatment can improve outcomes and prevent primary stroke. Multiple barriers exist, including limited diagnostic screening, inconsistent availability of and access to hydroxyurea, and knowledge gaps. These barriers are influenced by social, economic and policy factors that can be addressed to build capacity and improve outcomes.

## Introduction

HbSS disease, also known as sickle cell anemia (SCA), is an inherited hematological disease characterized by severe anemia, susceptibility to infection, recurrent painful episodes, and progressive organ failure. SCA is associated with increased morbidity and mortality, particularly in children under 5 years of age, and most significantly impacts resource-limited settings where capacity for diagnosis, screening for complications, and treatment is limited. Between 2000 and 2021, births of infants with SCA increased by 13.7% worldwide, and mortality due to SCA was 11 times higher than that of all-cause mortality [1]. The mortality burden of SCA is highest in children, particularly in countries with the highest under-five mortality rates, with SCA ranking in the top 20 causes of death in sub-Saharan Africa as compared to 76th −137th in respective high-income countries [1].

Given the substantial global burden of SCA, major changes in healthcare education and training are needed to improve management and treatment in low-resource settings. The spectrum of SCA care is both broad and deep, and to be comprehensive would include proper diagnosis using either standard laboratory tests or point-of-care devices; early intervention with prophylactic antibiotics such as penicillin by 3 months of age; complete childhood immunizations including pneumococcal vaccines; acute clinical complications including pain, pneumonia, and stroke; and chronic organ dysfunction due to progressive ischemic injury. It is paramount to focus on primary stroke, an important clinical complication, and the use of oral hydroxyurea, the only available disease-modifying treatment.

Primary stroke, which is defined as the first stroke in an individual patient, is a well-known and devastating complication of SCA, occurring in children as young as 2 years of age. Publications from before routine screening and treatment of primary stroke became best practice provide natural history data regarding the frequency and severity of stroke. Children between the ages of five and nine have the highest incidence, and by the age of 20, the cumulative incidence of primary stroke is 11%, with up to one-third of patients dying as a result [2]. Those who survive their first stroke experience a variety of neurocognitive disabilities, and recurrent stroke is common especially in the first 3 years following the initial incident [2]. The substantial morbidity and mortality associated with stroke in this population exemplify the need for the implementation of locally feasible screening methods that allow children at increased risk to be identified and life-saving preventive treatment to be initiated.

Hydroxyurea is a simple, potent disease-modifying medication for SCA that increases fetal hemoglobin (HbF), inhibits intracellular sickling, limits vaso-occlusion, and reduces hemolysis and inflammation [3]. Hydroxyurea has been shown to be safe and effective as a once-daily oral therapy that improves clinical outcomes in sub-Saharan Africa [4, 5]. Its utilization is limited in most pediatric settings in sub-Saharan Africa, and strategies to achieve optimal dosing require frequent clinic visits and laboratory monitoring, which present feasibility challenges in resource-limited settings. Screening children for risk of complications in conjunction with improved access to hydroxyurea represents an opportunity to improve capacity in resource-limited settings that can be bolstered through North-South partnerships.

The primary objective of this review is to describe how public health leaders can most effectively leverage screening tools such as transcranial Doppler (TCD) to identify children with SCA who are at increased risk of primary stroke in resource-limited settings in East and Central sub-Saharan Africa. While screening for retinopathy, pulmonary hypertension, silent cerebral infarcts, and nephropathy is recommended according to the United States National Heart, Lung, and Blood Institute guidelines and published data, these are beyond the scope of this work [6].

This review aims to 1) understand the magnitude of the problem; 2) gain further insight into TCD as an example of an effective, feasible mechanism to identify children with SCA who are at risk for primary stroke; and 3) understand the operational and systemic factors that impact the training and implementation of screening tools and hydroxyurea treatment in a resource-limited setting. Secondary review objectives included describing where children with SCA seek care in resource-limited settings, including healthcare infrastructure, cultural issues, and technology transfer, and how public health change models can be applied to understand and define a pathway for change.

## Methods

A narrative literature review design is a comprehensive, critical and objective analysis of the current published knowledge on a specific topic that is used to facilitate the establishment of theoretical frameworks and provide context for research [7]. This design was selected *in lieu* of a systematic review due to the breadth of the topic and the need to address several secondary questions within a complex, multidimensional paradigm of medicine, public health policy and global health infrastructure. Inclusion and exclusion criteria, search terms and an extraction table were included in the methods to add rigor to the results by utilizing key components of the systematic review model [8] while allowing for the flexibility of the narrative approach, which is more suited to thematic analysis and the development of theoretical models in a global health setting.

### Search Strategy

Given that the care of children with SCA in resource-limited settings and TCD screening for primary stroke in this context has been studied in multiple disciplines including medicine, science, public health, and international public health, a narrative, thematic strategy was employed to review qualitative and quantitative research. Peer-reviewed articles were the primary source of literature to provide background, significance, and overall context. A comprehensive literature review included background information on SCA; its clinical characteristics, course, and outcomes with and without treatment; available treatment options; and social, economic, and political factors influencing access to care in resource-limited settings in East and Central Africa.

Consideration was given to data from the United States as compared to data from sub-Saharan Africa, and a focused review of available sources of literature specific to healthcare settings in East and Central sub-Saharan Africa was performed. Further contextual information was included to provide appropriate background on TCD as a clinical screening tool for primary stroke, healthcare infrastructure within the identified settings, capacity gaps, and leadership change models relevant to the research objectives and context. Manuscripts published in peer-reviewed journals, such as *Lancet*, *Lancet Global Health*, *New England Journal of Medicine*, *Blood*, and *British Journal of Haematology*, were referenced; however, impact factor was considered secondary to content, robustness of the findings, and relevance to the population and locations identified. Recurrent stroke, which involves a different level of risk and complications, was not the focus of this review.

### Keywords and Search Terms

Keyword searches of the data sources (Table 1) were based on key concepts relevant to the primary and secondary research questions and utilized common synonyms and terminology to broaden the scope of the results. The results were reviewed to further refine the criteria to access the most relevant literature. Keyword searches included relevant terms in both the American and British English languages, such as anemia/anaemia and hemoglobin/haemoglobin, given the common use of both spelling formats in the project settings (Table 2).

**TABLE 1:** Literature sources (United States, Europe, East and Central Africa, 1990–2024).

Data Sources	Search	Type
Medline Full Text (EBSCO)	Sickle cell disease OR sickle cell anemia AND hydroxyurea; sickle cell disease AND morbidity OR mortality, sickle cell anemia AND stroke AND transcranial Doppler OR hydroxyurea	Peer-reviewed journal articles
Medline (PubMed)	Same as above	Peer-reviewed journal articles
Google Scholar	Sickle cell disease AND [Uganda, Ghana, Tanzania, Angola, Kenya or DRC], hemoglobinopathies, sickle cell disease AND sub-Saharan Africa AND East and Central Africa OR healthcare capacity gaps; change management AND sub-Saharan Africa AND Re-Aim OR Collective Impact Model OR technology transfer	Peer-reviewed journal articles, policy statements
World Health Organization	Prevalence of sickle cell disease and [Uganda, Tanzania, Angola, Kenya or DRC] AND public health AND sickle cell disease	Dataset, reports, policy statements/directives
IAHO (Integrative African Health Observatory)	Healthcare, pediatrics, sickle cell disease	Datasets

**TABLE 2:** Sample Keywords and search terms (United States, Europe, East and Central Africa, 1990–2024).

Concept	Synonyms or Related Terms
Sickle cell disease	Sickle cell anemia, sickle cell anaemia, HbSS, Hemoglobin S disease, drepanocytosis
Hydroxyurea	Hydroxycarbamide, Siklos^®^, Hydrea^®^, Droxia^®^
Pediatric	Children, adolescents, paediatric
Transcranial Doppler	TCD, ultrasound screening for primary stroke
Hemoglobinopathy	Red cell disorders, inherited hemoglobinopathy
Prevalence	Incidence, disease burden, and cumulative incidence
Capacity building	Training, grassroots efforts, development, education, healthcare infrastructure development, workforce development, North-South partnerships
Leadership	Leaders, stakeholders, decision-making, authority
Treatment	Therapies, interventions, supportive care, preventative care, prophylaxis (as penicillin, immunizations, etc.)
Morbidity and mortality	Death, clinical complications, and clinical outcomes
Feasibility	Suitability, viability
Barriers	Obstacles, limitations

### Inclusion and Exclusion Criteria

The criteria used to select manuscripts for analysis included peer-reviewed journal articles or official policy statements from the World Health Organization (WHO), ministries of health in sub-Saharan Africa, or similar health policy agencies that were published between 01 January 1990 and 30 June 2024. The primary focus of the literature was sickle cell anemia and/or public health and governance in resource-limited settings. Quantitative studies including phase 1–4 interventional clinical trials, epidemiologic studies, prospective cohort studies, and/or ancillary studies and qualitative studies such as surveys, interviews, and in-person observations were eligible for inclusion. Preference was given to clinical trials conducted in East and Central sub-Saharan Africa for greater relevance to the population. Editorials and media coverage were included only if directly related to the research questions. Studies were excluded from the review if published before 01 January 1990 or if they were clinical trials that only included adult participants.

### Patient and Public Involvement

Patients and the public were not involved in any way while conducting this literature review.

## Results

### Results of the Study Selection

**FIGURE 1:**
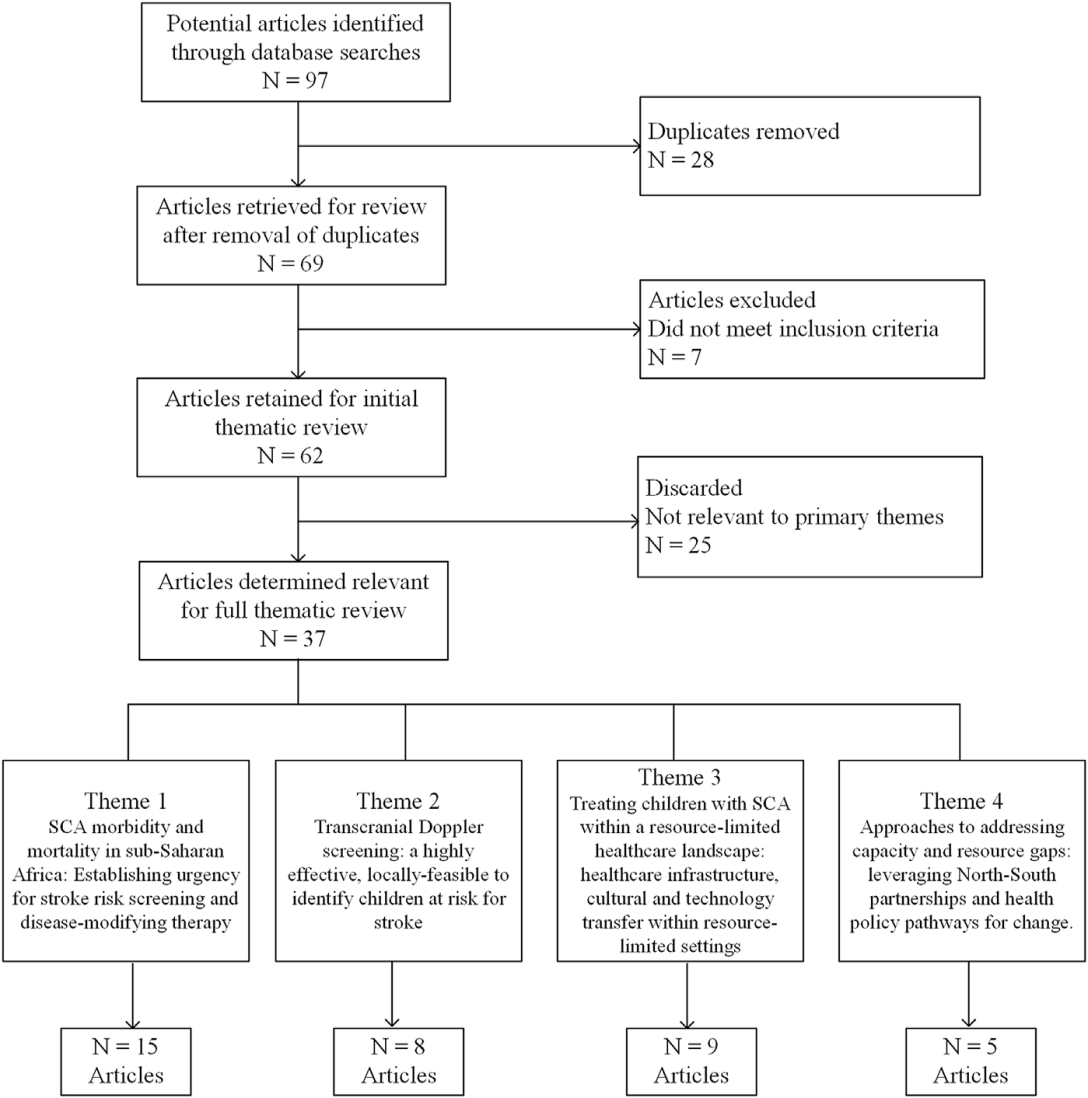
Literature review CONSORT diagram (United States, Europe, East and Central Africa, 1990–2024).

Initial searches returned a total of more than 90 manuscripts. This list was further refined to 37 publications that were categorized into four main themes, (1) the morbidity and mortality of SCA in East and Central sub-Saharan Africa, (2) literature supporting TCD as an effective and feasible tool to identify primary stroke risk in children with SCA, (3) current knowledge and resource/infrastructure gaps relevant to SCA in a resource-limited healthcare landscape, and (4) approaches to address capacity and resource gaps that leverage North-South partnerships and health policy pathways, as shown in Figure 1. These were then analyzed into relevant secondary themes. A full summary of the results in the form of an extraction table is included in Supplemental Appendix SA.

### Key Themes Identified in the Literature

#### Morbidity and Mortality of SCA in Sub-Saharan Africa: Establishing the Urgency of Stroke Risk Screening and Disease-Modifying Therapy

SCA is a common and life-threatening hematological condition affecting the development of red blood cells which results in significant clinical morbidity and mortality. Ware et al. described a wide range of severe clinical complications reflecting the complex pathophysiology of the condition, including vaso-occlusive painful crises, infection, anemia, and catastrophic organ damage, noting that these are rarely fatal in well-resourced countries [9]. Outcomes were found to be worse in resource-limited settings where healthcare infrastructure was limited. Barriers included a lack of clinical knowledge, delayed diagnosis, limited access to preventative care such as immunizations, penicillin prophylaxis and malaria prophylaxis, scarce and/or unsafe blood supplies, and limited access to disease-modifying therapy.

McGann et al. noted that SCA affects nearly every organ in the body, with acute and chronic disease manifestations due to the critical role of hemoglobin in delivering oxygen to all organs [10]. In the United States, mortality in children under 5 years of age with SCA was found to have decreased significantly due to diagnosis through universal newborn screening programs and interventions such as penicillin prophylaxis and pneumococcal vaccination [11]. In resource-limited settings, therapeutic options were found to be primarily limited to packed erythrocytes or exchange blood transfusions. In sub-Saharan Africa, transfusion use is limited by major challenges including inadequate blood supply, a high risk of transfusion-transmitted infections, and the risk of transfusion reactions [12].

The WHO (2010) noted that in many African countries, an estimated 10%–40% of the population carries the sickle cell gene resulting in an estimated disease prevalence of at least one to two percent [13]. In a multicenter, retrospective case-control study, Ranque et al. found mortality rates for children with SCA of 15.6% in those younger than 1 year of age, 36.4% in children under 5 years, and 43.3% in children less than 10 years of age [14]. The 2021 Global Burden of Disease analysis further documented that SCA contributes significantly to all-cause mortality in pediatric populations and underscored the importance of surveillance efforts and research to inform the deployment of evidence-based prevention and treatment of SCA [1].

Uganda was one of the first countries in Africa with a documented SCA disease burden. A surveillance study of infants born to HIV-positive mothers whose blood had been collected at birth confirmed the high prevalence of sickle cell trait and SCA within the country, with notable variation between regions and districts [15]. These data have informed further research and national strategies in Uganda and other sub-Saharan African countries, including neonatal screening, clinical screening, and treatment interventions in targeted high-risk areas.

Primary stroke is one of the most serious acute complications of SCA with devastating consequences for patients and their families and a significant burden on healthcare systems in resource-limited settings. A systematic review found that the prevalence of stroke in children with SCA living in sub-Saharan Africa ranged from 2.9% to 16.9%, with disease mortality, inaccurate diagnosis and regional variability limiting more precise estimates [16]. The authors argued that high disease- and stroke-related mortality led to an underestimation of the number of children affected by stroke. Stroke in SCA occurs primarily in childhood, with the sudden onset of neurological dysfunction due to restricted or fully obstructed blood flow that inhibits delivery of oxygen to the intracranial arteries, necessitating immediate intervention through exchange transfusion [10]. Clinical consequences of neurological complications include death, lifelong physical and cognitive disability, special education needs, and increased risk of recurrent stroke [17]. These complications are more difficult to manage in resource-limited settings, emphasizing the importance of screening tools such as TCD to identify children at risk and the utilization of hydroxyurea at an optimized dose to prevent stroke in this population.

#### Transcranial Doppler (TCD): A Feasible, Effective Method to Identify Children at Risk for Stroke

Primary stroke in SCA represents a risk that can be mitigated with appropriate screening and preventive treatment. TCD is a screening method that uses non-invasive ultrasonography to measure the velocity of blood in the brain and detect stenosis by identifying vessels with high arterial flow velocities [18]. The highest time-averaged mean velocity (TAMV) measured in three major cerebral arteries has become the reported standard value for clinical decision-making in children with SCA [19]. Adams et al. demonstrated in a landmark study that TCD could be effectively used to screen children with SCA for primary stroke risk [18], showing that the relative risk of stroke was more than 40 times greater in children with a TAMV of ≥200 cm/s. This was the basis for a subsequent prospective randomized clinical trial in which children at high risk for stroke were treated with blood transfusions, after which many pediatric sickle cell centers in the United States and Europe initiated TCD screening to identify and treat children with SCA at high risk for the development of primary stroke [20].

TCD is an attractive tool in resource-limited settings due to its relative ease of performance, lack of need for sedation, and lack of radiation exposure. It is non-invasive, painless, and portable, and its results can be reproduced when performed and scored using appropriate standards. Due to intra-individual variation in TCD velocities over time, serial TCD measurements are typically performed as part of a clinical TCD screening program which underscores the importance of defined standard operating procedures, training methodology, and examination review to minimize variability and improve reliability, validity, and clinical decision-making based on TCD results [21].

TCD screening is relevant in settings where universal access to disease-modifying therapy is limited due to cost, availability, or other factors. Identifying children at the highest risk for primary stroke and facilitating access to treatment has the potential to dramatically improve clinical outcomes in this high-risk group while also reducing the long-term burden on families and healthcare systems. A prospective, open-label study of TCD screening in a cohort of children with SCA in Tanzania identified a subset with elevated velocities who were treated with hydroxyurea with escalation to MTD. A rapid, substantial, and sustained reduction in TCD velocities and reduced risk of stroke was observed in the study population along with improved laboratory and clinical outcomes [22].

A barrier in all clinical settings is the lack of skilled personnel trained to administer TCD exams. Without adequate technical training and oversight, the reliability and validity of TCD results are limited. There is poor inter-rater reliability between untrained TCD examiners and those who have undergone structured training programs, with higher velocities identified in untrained examiners and greater sensitivity to TCD results in trained operators [23]. A 2020 multicenter assessment of TCD training in Europe demonstrated that this obstacle can be mitigated through the implementation of a standardized and reproducible training program that utilizes competency validation. The authors found that the use of imaging TCD was superior to non-imaging TCD and required less training to reach competency; however, non-imaging TCD may represent a more cost-effective and logistically sound option in resource-limited settings in sub-Saharan Africa [24].

#### Treating Children With SCA in a Resource-Limited Healthcare Landscape: Healthcare Infrastructure, Cultural and Technology Transfer in Resource-Limited Settings

Multilayer capacity gaps exist that lead to a lack of understanding of SCA at the provider and patient/family level in sub-Saharan Africa, resulting in reduced access to screening, diagnosis, and life-saving care. These include misconceptions about SCA, the cost of screening and treatment, and inadequate healthcare infrastructure to provide continuity of care across the lifespan. In addition to the morbidity and mortality resulting from complications of SCA, the disease negatively impacts individual and family quality of life, increases healthcare utilization and strains resource-limited healthcare systems. In sub-Saharan Africa, the disease burden is exacerbated by inadequate healthcare infrastructure, poor nutrition and infectious comorbidities including malaria, tuberculosis, and HIV [25].

Research on the disease burden of SCA and the availability of safe and effective disease-modifying treatment options in East and Central sub-Saharan Africa suggests that with the appropriate public health policies and improved education among patients, providers and communities, the outcomes of children with SCA in these settings have the potential to improve substantially. A survey study of 1,829 participants with SCA in eastern Uganda found that approximately half of the participants knew that SCA was inherited from both parents, but a substantial proportion did not know how the disease was transmitted, many of them believing that SCA was transmitted by blood transfusion [26]. Furthermore, less than 10% were taking hydroxyurea, 20% reported feeling stigmatized, and 80% reported being hospitalized for SCA-related complications in the past year, all of which underscore the need for improved care for SCA patients in Uganda.

If TCD can be effectively implemented in these settings to screen for primary stroke risk, clinicians will need to have available treatment options for at-risk patients, and policymakers will need to support health systems to address the many knowledge gaps that exist in this area. Hydroxyurea has been shown to be a safe, accessible, and effective disease-modifying therapy that improves clinical outcomes in children with SCA [4, 27, 28]. Its utilization is limited in most pediatric settings in sub-Saharan Africa, and conventional strategies to achieve an appropriate, optimal dose require frequent clinic visits and laboratory monitoring, which pose feasibility challenges in resource-limited settings. Recently, escalation of hydroxyurea to MTD has been proven to be superior for reducing complications of SCA compared to a lower, fixed dose [5, 29] and has reduced the incidence of blood transfusions in affected patients [30, 31].

Hydroxyurea has a relatively narrow therapeutic index and works best at an optimal dose. The optimal dose differs by individual although the majority of patients require more than the traditional 20 mg/kg/day. While escalation to higher optimal doses has been shown to be safe and effective, multiple barriers exist to achieving optimal dosing via standard dose escalation outside of clinical trials. Dose escalation is a resource-intensive process that takes 6-12 months and requires frequent clinic visits to make dose adjustments and accurate laboratory monitoring to avoid dose-limiting toxicities [29]. In order to balance achieving maximal efficacy and limiting toxicity, this process must often be supervised by a trained hematologist or pediatrician, which is limited by a shortage of trained providers, suggesting opportunities for systematic improvement of dosing strategies from an educational, systemic, or technology transfer perspective to make the treatment more accessible in local healthcare settings.

With regard to neurological protection and the mitigation of morbidity and mortality associated with primary stroke risk, a prospective phase 2 open-label trial involving children with SCA and documented cerebral vasculopathy living in a resource-limited Caribbean setting showed that hydroxyurea treatment escalated to MTD significantly reduced TCD velocities, provided laboratory and clinical benefits, and offered primary stroke prevention [32]. With evidence of decreased stroke risk statistically evident, the fact that the treatment was also found to be safe between risk groups further supports the potential benefits of its utilization, particularly in resource-limited settings where access to a consistent and safe blood supply for transfusion is limited.

#### Approaches to Addressing Capacity and Resource Gaps: Leveraging North-South Partnerships and Health Policy Pathways for Change

Human resources limitations in healthcare settings, exacerbated by limited healthcare infrastructure and insufficient health policies to prioritize limited resources to areas of high impact are a major challenge in Africa. There are inequities in access to healthcare services at all levels of care, which were evident before and have been exacerbated after the COVID-19 pandemic [33]. Healthcare providers may have limited clinical training and evidence-based knowledge, which magnifies existing challenges in screening and treatment, providing an opportunity for capacity building and knowledge transfer in a meaningful, sustainable manner from well-resourced to resource-limited settings. Frenck et al. supported this barrier of “collective failure” in the equitable transfer of healthcare advances and scientific knowledge between well-resourced and resource-limited settings [34]. TCD training is an example of an opportunity to improve clinical skills and implement clinical decision-making tools through distance and in-person education as a realistic step to facilitate change in this area.

The literature emphasizes the importance of the Ministry of Health’s involvement in continuously sensitizing the public to the facts about SCA to mitigate the effects of these misconceptions that limit the health-seeking behaviors of patients and families [35]. As access to basic care and treatment increases for children with SCA in the United States and Europe, the increasingly widening gap in sub-Saharan Africa represents a major health disparity that can be mitigated through effective implementation of locally feasible tools such as TCD, which can be appropriately scaled and aligned with increased availability of treatment options through health policy support and stakeholder engagement to drive change and improve outcomes in this underserved population.

## Discussion

The results of this review indicate the substantial morbidity and mortality associated with SCA in East and Central sub-Saharan Africa and the critical need for improved access to screening and improved access to hydroxyurea to prevent early mortality and clinical morbidity in this setting. The morbidity and mortality associated with SCA in sub-Saharan Africa are crucial to provide context for the magnitude of the problem and the urgency of the overall need for improved diagnosis, screening for complications and treatment of affected children. Stroke is an example of a significant risk in this population that can be mitigated through the implementation of locally accessible tools with appropriate support for interpretation and clinical decision-making. TCD ultrasonography represents an evidence-based screening tool that can be used to mitigate serious clinical complications of stroke in children with SCA, which is of particular relevance in resource-limited settings. Moreover, this review outlined current knowledge and resource-infrastructure gaps and proposed approaches to address these gaps by leveraging North-South partnerships and impactful health policies.

The role of TCD as a screening tool for primary stroke in SCA necessitates a description of available treatments with a specific focus on resource-limited settings, including the relationship between healthcare infrastructure, cultural issues, technology, and knowledge transfer. The background of TCD screening was explored, including a review of research that has validated its use as a screening tool in the population of children with SCA in the United States and the gaps that exist in training methodology outside of the global North. These can be used to further assess needs in resource-limited settings and develop training methodologies that can be effectively applied to meet local needs. Leveraging North-South partnerships and health policy pathways is a key theme that is directly linked to implementing change to build capacity within healthcare systems in resource-limited settings, utilizing public health leadership models and strategies to facilitate sustainable policy and systems change. The efficacy of TCD screening and the benefits of hydroxyurea use for children with SCA living in sub-Saharan Africa have been clearly documented but prospective research is needed to identify and implement effective, accessible methods of TCD screening in local settings.

More broadly, the literature has demonstrated the safety and efficacy of hydroxyurea as a disease-modifying therapy for SCA with a direct impact on stroke risk at optimal doses; however, there is a large gap that exists between the implementation of TCD screening and the accessibility of hydroxyurea to a broader population of patients. Therefore, it is necessary to understand the factors that underlie these gaps, along with the potential mechanisms to mitigate them. This relatively broad theme is inclusive of the local context in addition to specific medical, technological, and public health-related opportunities that can be explored to improve hydroxyurea utilization, and the role of leadership and change management to support capacity building through the expansion of research and other programmatic partnerships between the United States and sub-Saharan African countries. These findings are consistent with previous reviews making the case for improved capacity to diagnose, screen and treat SCA in resource-limited settings [17, 36, 37]. Our review builds on these findings by synthesizing key themes that can be utilized to make recommendations regarding the use of North-South partnerships to address educational, technology transfer and health policy gaps, which collectively constitute an opportunity for capacity building to improve outcomes for SCA in resource-limited settings.

The goal of furthering research and improving clinical care globally necessitates the development of long-term partnerships between research groups working on SCA in well-resourced and resource-limited countries [35]. These partnerships facilitate the transfer of knowledge and technology to improve local healthcare infrastructure and develop the healthcare workforce and represent an opportunity to implement lasting change beyond the research settings when the local context is considered. Use of leadership change models to understand the local context and to establish a structured methodology for change through North-South partnerships is an effective strategy that utilizes the strengths and resources of the parties involved toward a common set of goals. The Re-AIM and Collective Impact leadership change models are relevant to understanding the complex issues associated with implementing change in healthcare systems and informing policy in resource-limited settings.

The Re-AIM model was developed to assist in the planning and evaluation of clinical and community-based projects and can be used as a tool in the translation of research into practice. The model utilizes a multidimensional approach that includes reach, efficacy, adoption, implementation, and maintenance to support a structured and measurable approach to change management relevant to public health programming and policy [37]. This model translates well into international practice due to its use of core dimensions that allow for a multifaceted understanding of implications in real-world settings and provide a useful lens through which programs such as TCD screening and training can be defined in local contexts [38].

The Collective Impact Model calls for individuals, organizations, and entities to abandon individual agendas in favor of a single, common agenda, requiring all participants to develop a common understanding of a health system problem and a mutual agreement to solve it. This is particularly relevant to public health leadership change management given the multiple stakeholders, roles and disciplines involved in international health systems all of whom need to change behaviors to solve complex public health problems [39]. Shifting from isolation within groups to the context of a broader system has a direct and indirect impact on how practitioners design and implement work processes, how funders incentivize and engage with workers and program teams, and how policymakers bring solutions to a broader scale [40].

### Limitations

The review has focused on East and Central Africa, which together have an enormous burden of SCA according to the Global Burden of Disease publications [1]. By necessity, substantial published work from Northern Africa, West Africa, and South Africa was not included. Similarly, resource-limited settings in Asia, the Middle East, and the Caribbean were not included. The intended focus on stroke prevention and exclusion of a detailed review of other screening strategies for patients with SCA was made for practical reasons related to the volume and scope of this review. Furthermore, this review focused primarily on published clinical trial data and was therefore limited to the inclusion of qualitative research that may influence the public health context and associated implementation strategies in sub-Saharan Africa. The focus on hydroxyurea as a therapeutic option did not include a comparative analysis of the literature on other possible treatment options such as bone marrow transplantation or gene therapy which may be of clinical relevance.

### Conclusion

The complex landscape of SCA in the context of sub-Saharan Africa is clearly described by this literature review, which indicates that SCA represents a significant public health challenge in East and Central Africa. Despite advancements in the diagnosis and management of SCA, multiple barriers to optimal clinical care exist, including well-defined stroke screening programs utilizing TCD, limited availability and access to hydroxyurea, and knowledge gaps among the healthcare workforce that necessitate a multifaceted approach to capacity building in resource-limited settings. While working to improve care within a healthcare and research context in these settings involves inherent challenges, it also provides an opportunity for collaboration with local communities to identify and operationalize the change management strategies that are most likely to achieve their goals. By engaging in a research partnership with local medical and public health leaders as well as government agencies, community groups, and other key stakeholders, public health change management steps can be implemented in an approach that is locally relevant, culturally appropriate, and scientifically sound. The literature supporting the themes identified in this review demonstrates the significant disease burden of SCA in this population and the extensive impact that research partnerships can have to improve outcomes for this underserved, at-risk population.
