# Supplementary material for: Primary Stroke Screening and Hydroxyurea Treatment for Sickle Cell Anemia in Pediatric Healthcare Settings in East and Central Africa: A Narrative Review of Capacity Gaps and Opportunities
Source: Public Health Rev. 2025 May 15;46:1608359. doi: 10.3389/phrs.2025.1608359 (PMC12119301; doi:10.3389/phrs.2025.1608359)
Supplement: Supplementary file 1 [file Table1.DOCX]

Supplemental Appendix: Extraction Table

| **Author** | **Title** | **Publication Information**  **(Journal, Year)** | **Area of Focus/Primary Theme** |
| --- | --- | --- | --- |
| Adams, R. | The use of transcranial ultrasonography to predict stroke in sickle cell disease | The New England Journal of Medicine, 1992 | Transcranial Doppler screening: a highly effective, locally feasible to identify children at risk for stroke |
| Adams, R. | Prevention of a first stroke by transfusions in children with sickle cell anemia and abnormal results on transcranial Doppler ultrasonography | The New England Journal of Medicine, 1998 | Transcranial Doppler screening: a highly effective, locally feasible to identify children at risk for stroke |
| Ali, S. | Stroke recurrence in children with sickle cell disease treated with hydroxyurea following first clinical stroke | American Journal of Hematology, 2011 | SCA morbidity and mortality in sub-Saharan Africa: Establishing urgency for stroke risk screening and disease-modifying therapy |
| Ambrose, E. | Hydroxyurea with dose escalation for primary stroke risk reduction in children with sickle cell anaemia in Tanzania (SPHERE): An open-label, phase 2 trial | The Lancet Haematology, 2023 | Transcranial Doppler screening: a highly effective, locally feasible to identify children at risk for stroke |
| Aygun, B. | Hydroxyurea dose optimisation for children with sickle cell anaemia in sub-Saharan Africa (REACH): extended follow-up of a multicentre, open-label, phase 1/2 trial | The Lancet Haematology, 2024 | SCA morbidity and mortality in sub-Saharan Africa: Establishing urgency for stroke risk screening and disease-modifying therapy |
| Bhuiyan, M.R. | The effect of formal training on the clinical utility of transcranial Doppler ultrasound monitoring in patients with aneurysmal subarachnoid haemorrhage | Journal of Clinical Neuroscience, 2012 | Transcranial Doppler screening: a highly effective, locally feasible to identify children at risk for stroke |
| Brambilla, D.J. | Intra-individual variation in blood flow velocities in cerebral arteries of children with sickle cell disease | Pediatric Blood & Cancer, 2007 | Transcranial Doppler screening: a highly effective, locally feasible to identify children at risk for stroke |
| Egesa, W.A. | Sickle cell disease in children and adolescents: A review of the historical, clinical, and public health perspective of sub-Saharan Africa and beyond | International Journal of Pediatrics, 2022 | Treating children with SCA within a resource-limited healthcare landscape |
| Frenk, J. | Health professionals for a new century: Transforming education to strengthen health systems in an interdependent world | The Lancet, 2010 | Treating children with SCA within a resource-limited healthcare landscape |
| Glasgow, R.E. | Evaluating the public healt impact of health promotion interventions: The RE-AIM framework. | American Journal of Public Health, 1999 | Approaches to addressing capacity and resource gaps: leveraging North-South partnerships and health policy |
| Glasgow, R.E. | Pragmatic applications of RE-AIM for health care initiatives in community and clinical settings | Preventing Chronic Disease, 2018P | Approaches to addressing capacity and resource gaps: leveraging North-South partnerships and health policy |
| Inusa, B.P.D | An educational study promoting the delivery of transcranial Doppler ultrasound screening in paediatric sickle cell disease: A European multi-centre perspective | Journal of Clinical Medicine, 2019 | Transcranial Doppler screening: a highly effective, locally feasible to identify children at risk for stroke |
| John, C.C. | Hydroxyurea dose escalation for sickle cell anemia in sub-Saharan Africa | JThe New England Journal of Medicine, 2020 | SCA morbidity and mortality in sub-Saharan Africa: Establishing urgency for stroke risk screening and disease-modifying therapy |
| Kania, J. | Collective Impact | Stanford Social Innovation Review, 2011 | Approaches to addressing capacity and resource gaps: leveraging North-South partnerships and health policy |
| Kania, J. | Essential mindset shifts for collective impact | Stanford Social Innovation Review, 2013 | Approaches to addressing capacity and resource gaps: leveraging North-South partnerships and health policy |
| Lobo, C. | Hydroxyurea therapy reduces mortality among children with sickle cell disease | Blood, 2010 | SCA morbidity and mortality in sub-Saharan Africa: Establishing urgency for stroke risk screening and disease-modifying therapy |
| Marks, L.J. | Stroke prevalence in children with sickle cell disease in sub-Saharan Africa: A systematic review and meta-analysis | Global Pediatric Health, 2018 | SCA morbidity and mortality in sub-Saharan Africa: Establishing urgency for stroke risk screening and disease-modifying therapy |
| Mburu, J. | Sickle cell disease: Reducing the global disease burden | International Journal of Laboratory Hematology, 2019 | Treating children with SCA within a resource-limited healthcare landscape |
| McGann, P.T. | Sickle cell anemia in sub-Saharan Africa: Advancing the clinical paradigm through partnerships and research | Blood, 2017 | SCA morbidity and mortality in sub-Saharan Africa: Establishing urgency for stroke risk screening and disease-modifying therapy |
| McGann, P.T. | Clinical features of β-thalassemia and sickle cell disease | Gene and cell therapies for beta-globinopathies, 2017 | SCA morbidity and mortality in sub-Saharan Africa: Establishing urgency for stroke risk screening and disease-modifying therapy |
| Moodley, K. | Allocation of scarce resources in Africa during COVID-19: Utility and justice for the bottom of the pyramid? | Developing World Bioethics, 2021 | SCA morbidity and mortality in sub-Saharan Africa: Establishing urgency for stroke risk screening and disease-modifying therapy |
| Ndeezi, G | Burden of sickle cell trait and disease in the Uganda sickle surveillance study (US3): A cross-sectional study | The Lancet Global Health, 2016 | SCA morbidity and mortality in sub-Saharan Africa: Establishing urgency for stroke risk screening and disease-modifying therapy |
| Nichols, F.T. | Stroke prevention in sickle cell disease (STOP) study guidelines for transcranial Doppler testing | Journal of Neuroimaging, 2001 | Transcranial Doppler screening: a highly effective, locally feasible to identify children at risk for stroke |
| Ohene-Frempong, K. | Cerebrovascular accidents in sickle cell disease: Rates and risk factors | Blood, 1998 | Treating children with SCA within a resource-limited healthcare landscape |
| Olupot-Olupot, P. | Characterizing demographics, knowledge, practices and clinical care among patients attending sickle cell disease clinics in Eastern Uganda | Wellcome Open Research, 2020 | Treating children with SCA within a resource-limited healthcare landscape |
| Power-Hays, A. | Hydroxyurea reduces the transfusion burden in children with sickle cell anemia: The REACH experience | Blood, 2021 | Treating children with SCA within a resource-limited healthcare landscape |
| Power-Hays, A. | Reducing transfusion utilization for children with sickle cell anemia in sub-Saharan Africa with hydroxyurea: Analysis from the phase I/II REACH trial | American Journal of Hematology, 2024 | Treating children with SCA within a resource-limited healthcare landscape |
| Quinn, C.T. | Improved survival of children and adolescents with sickle cell disease | Blood, 2010 | SCA morbidity and mortality in sub-Saharan Africa: Establishing urgency for stroke risk screening and disease-modifying therapy |
| Rankine-Mullings, A. | Hydroxycarbamide treatment reduces transcranial Doppler velocity in the absence of transfusion support in children with sickle cell anaemia, elevated transcranial Doppler velocity, and cerebral vasculopathy: the EXTEND trial | British Journal of Haematology, 2021 | Transcranial Doppler screening: a highly effective, locally feasible to identify children at risk for stroke |
| Ranque, B. | Estimating the risk of child mortality attributable to sickle cell anaemia in sub-Saharan Africa: A retrospective, multicentre, case-control study | The Lancet Haematology, 2022 | SCA morbidity and mortality in sub-Saharan Africa: Establishing urgency for stroke risk screening and disease-modifying therapy |
| Thomson AM | Global, regional, and national prevalence and mortality burden of sickle cell disease, 2000-2021: a systematic analysis from the global burden of disease study 2021 | The Lancet Haematology, 2023 | SCA morbidity and mortality in sub-Saharan Africa: Establishing urgency for stroke risk screening and disease-modifying therapy |
| Tshilolo, L. | Hydroxyurea for children with sickle cell anemia in sub-Saharan Africa | The New England Journal of Medicine, 2019 | SCA morbidity and mortality in sub-Saharan Africa: Establishing urgency for stroke risk screening and disease-modifying therapy |
| Wang, W.C. | Hydroxycarbamide in very young children with sickle-cell anaemia: A multicentre, randomised, controlled trial (BABY HUG) | The Lancet, 2011 | Treating children with SCA within a resource-limited healthcare landscape |
| Ware, R.E. | How I use hydroxyurea to treat young patients with sickle cell anemia | Blood, 2010 | Treating children with SCA within a resource-limited healthcare landscape |
| Ware, R.E. | Sickle cell disease | The Lancet, 2017 | SCA morbidity and mortality in sub-Saharan Africa: Establishing urgency for stroke risk screening and disease-modifying therapy |
| Weatherall, D. | A case for developing north–south partnerships for research in sickle cell disease | Blood, 2005 | Approaches to addressing capacity and resource gaps: leveraging North-South partnerships and health policy |
| World Health Organization | *Sickle-cell disease: A strategy for the WHO African region* | World Health Organization: Regional Office for Africa, (2010). | SCA morbidity and mortality in sub-Saharan Africa: Establishing urgency for stroke risk screening and disease-modifying therapy |
